# Elevation of Platelet and Monocyte Activity Markers of Atherosclerosis in Haemodialysis Patients Compared to Peritoneal Dialysis Patients

**DOI:** 10.1155/2017/8506072

**Published:** 2017-07-09

**Authors:** Ksenija Stach, Susanne Karb, Ibrahim Akin, Martin Borggrefe, Bernhard Krämer, Thorsten Kälsch, Anna-Isabelle Kälsch

**Affiliations:** ^1^1st Medical Department, University Medical Centre Mannheim, Medical Faculty Mannheim, University of Heidelberg, Heidelberg, Germany; ^2^5th Medical Department, University Medical Centre Mannheim, Medical Faculty Mannheim, University of Heidelberg, Heidelberg, Germany; ^3^Herzzentrum Weinheim, University Medical Centre Mannheim, Medical Faculty Mannheim, University of Heidelberg, Heidelberg, Germany

## Abstract

**Purpose:**

The predominant cause of mortality in dialysis patients are cardiovascular events. Platelet and monocyte activity markers play an important role in cardiovascular mortality and were assessed and related to dialysis quality criteria in haemodialysis (HD) and peritoneal dialysis (PD) patients.

**Methods:**

For this prospective comparative study, HD patients (*n* = 41) and PD patients (*n* = 10) were included. In whole blood samples, surface expression of CD62P and CD40L on platelets, tissue factor binding on monocytes, and platelet-monocyte aggregates were measured by flow cytometry. Plasma levels of MCP-1, IL-6, TNF*α*, and soluble CD40L were analysed by enzyme-linked immunosorbent assay.

**Results:**

Haemodialysis patients showed a significantly higher CD62P expression on platelets (*p* = 0.017), significantly higher amount of platelet-monocyte aggregates (*p* < 0.0001), and significantly more tissue factor binding on monocytes (*p* < 0.0001) compared to PD patients. In PD patients, a significant correlation between Kt/V and platelet CD40L expression (*r* = 0.867; 0.001) and between Kt/V and platelet CD62P expression (*r* = 0.686; *p* = 0.028) was observed, while there was no significant correlation between Kt/V and tissue factor binding on monocytes and platelet-monocyte aggregates, respectively.

**Conclusion:**

Platelet and monocyte activity markers are higher in HD patients in comparison with those in PD patients, possibly suggesting a higher risk of cardiovascular morbidity and mortality.

## 1. Introduction

End-stage renal disease is associated with increased cardiovascular mortality and morbidity. The mortality rate in patients with end-stage renal disease (ESRD) is comparable to many cancers and is mostly caused by an increased mortality rate due to cardiovascular disease. A promising approach to reduce this mortality has been to increase dialysis dose. Therefore, possible correlations between dialysis dose and mortality have been studied in major trials with disappointingly negative results for both haemodialysis and peritoneal dialysis patients [1, 2]. Furthermore, increased circulating inflammatory proteins have been shown to predict a worse prognosis in ESRD patients [3].

Alterations in haemostasis are common complications of kidney diseases. Their frequency and severity correlate with the progressive loss of renal function to end-stage renal disease. Both bleeding diathesis and thromboembolic events have been identified [4]. The coagulation profile of patients with ESRD shows normal or elevated levels of coagulation factors, suggesting that occurrence of haemorrhagic events is mainly due to platelet dysfunction [5]. Proper haemostasis requires adhesion and aggregation of platelets at the site of vascular injury, as well as secretion of several adhesive substances by platelets [6]. The altered platelet function in ESRD is the result of impaired platelet adhesiveness as well as abnormal platelet endothelial interaction [7]. An interaction between activated platelets and endothelial cells plays an important pathophysiologic role in the development of atherosclerosis [8]. In detail, interactions between platelets and endothelial cells mediate essential processes in the development of atherosclerosis by an increased expression of vascular cell adhesion molecules and their ligands. These include P-selectin—an *α*-granule protein that mediates platelet rolling, leukocyte adhesion, and coagulation—tissue factor expression on monocytes, platelet-monocyte aggregates, and CD40L—a member of the tumor necrosis factor-*α* family of proteins [9]. CD62P and CD40L are expressed on activated platelets and are directly involved in the interaction of platelets with monocytes and endothelial cells [10].

The predominant cause of the mortality of dialysed patients are cardiovascular events related to thrombosis [11]. In this respect, the CD40 receptor and its ligand (CD40L) on activated platelets are of particular interest. They are known to modulate both inflammation and thrombosis, two processes important for the development and clinical expression of atherosclerosis [12]. Binding of CD40L to its CD40 receptor on endothelial cell membranes induces an enhanced release of potent proinflammatory and atherosclerosis-promoting cytokines and chemokines (e.g., IL-6 and MCP-1) [13]. Tissue factor expression on monocytes is important in extrinsic coagulation and elevated in HD patients compared to that in healthy controls [14]. It is known that a disturbed platelet function is an important cause for thromboembolic and haemorrhagic complications in ESRD patients.

Haemodialysis and peritoneal dialysis are both established methods in treating ESRD and aim to attenuate cardiovascular risk factors. In order to investigate possible functional differences between both dialysis modalities regarding and affecting atherothrombotic risk, the present study assessed platelet and monocyte activity markers of atherosclerosis and correlated these markers to dialysis dose in both dialysis modalities.

## 2. Methods

### 2.1. Study Population

For this prospective comparative study, haemodialysis patients (*n* = 41; 25 male, 16 female) or peritoneal dialysis patients (*n* = 10; 6 male, 4 female) were included consecutively.

The investigation conforms to the principles outlined in the Declaration of Helsinki. The study was approved by the local ethics committee and all patients gave informed consent to study participation and the use of their medical record for research purposes.

Haemodialysis patients were haemodialysed on hollow-fiber polysulfone membrane dialysators with a 1.3 m^2^ surface area (*n* = 40) (Polysulfone UF 5.5; Hemoflow F6, Fresenius, Germany) or with cellulose triacetate dialysator (*n* = 1) (CT 150 G, Baxter, Germany) with bicarbonate-containing solutions for on average 13.2 (9–20) hours weekly. Peritoneal dialysis solutions of 2 l with 1.36%, and when necessary 3.86%, glucose were used three or four times a day by the patients using peritoneal dialysis. All the patients were advised to comply with a chronic renal failure diet consisting of 35 kcal/kg, including 1.2/1.4 g/kg of protein, 1000/1500 mg of calcium, 700 mg of phosphorus, and 250 mg of magnesium, vitamins, and recombinant human erythropoietin. The Kt/V was 1.53 ± 0.28 for hemodialysis patients and 2.63 ± 0.65 for peritoneal dialysis patients.

### 2.2. Flow Cytometric Analysis

Blood samples of heparinized blood (15 IE heparin per ml blood) (Sarstedt AG & Co., Nuembrecht, Germany) were obtained. To avoid aggregation and activation of platelets, the blood samples were shaken over the time. All whole blood samples were subsequently investigated on activation of monocytes and platelets as well as platelets binding on monocytes.

Flow cytometric analysis of platelets was performed by gating in forward and side scatter. Platelets were gated back for determination of the expression of CD40L and CD62P. Platelet-monocyte aggregates were measured by CD41 (GPIIb/IIIa receptor) surface expression on platelets adherent to monocytes.

For the analysis of platelets, 100 *μ*l of each whole blood sample was stained for 30 min at room temperature with 10 *μ*l aliquots of mouse anti-human CD62P-PE antibodies (CLB-Thromb/6) (Coulter Immunotech, Krefeld, Germany) and mouse anti-human CD40L-FITC antibodies (P2) (Calbiochem/Merck KGaA, Darmstadt, Germany). For the analysis of monocytes, 100 *μ*l of each whole blood sample was stained for 30 min at room temperature with 10 *μ*l aliquots of PE-conjugated murine antibody against CD41 (Coulter Immunotech, Krefeld, Germany) and murine FITC-conjugated antibody against tissue factor (American Diagnostica, Pfungstadt, Germany). To identify monocytes, the probes were additionally stained with mouse anti-human CD14-ECD (RM052) (Coulter Immunotech, Krefeld, Germany). Isotype-matched mouse anti-human IgG1 PE/FITC antibodies (Beckman Coulter, Marseille, France) were used as a control. After incubation, erythrocytes were lysed with 500 *μ*l Optilyse C (Coulter Immunotech, Krefeld, Germany). After 15 min, cells were resuspended in 500 *μ*l PBS and were then ready for flow cytometric analysis. For measurement of CD62P and CD40L on platelets, a gating for forward and sideward scatter was performed. For measurement of CD41 on platelets adherent to monocytes to determine platelet-monocyte aggregates and for measurement of membrane-bound tissue factor, a gating for the monocyte surface antigen CD14 and sideward scatter was performed. All flow cytometric analysis was performed on an EPICS XL-MCL analyzer (Beckman Coulter, Krefeld, Germany) equipped with an argon laser tuned at 488 nm. Compensation of the four-channel fluorescence was precisely adjusted using Cyto-CompTM reagents and Cyto-TrolTM control cells (Coulter Immunotech, Krefeld, Germany). We followed the methods of Pirzer et al. 2012 [15].

### 2.3. Enzyme-Linked Immunosorbent Assay (ELISA)

Plasma concentrations of TNF*α* (Human TNF*α*/TNFSF1A Immunoassay R&D Systems, Inc., Wiesbaden, Germany), interleukin 6 (IL-6) (Human IL-6 Immunoassay R&D Systems, Inc., Wiesbaden, Germany), monocyte chemotactic protein-1 (MCP-1) (Human MCP-1/CCL2 Immunoassay R&D Systems, Inc., Wiesbaden, Germany), and CD40L (Human soluble CD40 Ligand Immunoassay R&D Systems, Inc., Wiesbaden, Germany) were determined by sandwich-type immunoassay according to the manufacturer instructions. All concentration analysis was performed on an ELISA-Reader-Lab Systems Multiskan RC (Lab Systems, Finland). Genesis Lite Software and ELISA Multiskan RC were used for data acquisition and evaluation.

### 2.4. Statistical Analysis

Numerical data were expressed as mean ± standard deviation (SD). A Mann–Whitney test was applied as a nonparametric test. Categorical variables were analysed using a chi-square test and *t*-test. Values are expressed as mean values ± SD. A two-tailed probability <0.05 was considered significant. All calculations were performed using GraphPad InStat version 3.01 (GraphPad Software, San Diego, California, USA) and SPSS Statistics version 17 (SPSS-Software GmbH, Munich, Germany).

## 3. Results

Baseline characteristics of the study patients as well as the medication use at baseline are given in Tables 1 and 2.

### 3.1. Difference between Haemodialysis and Peritoneal Dialysis regarding Platelet and Monocyte Activity Markers

**Figure 1:**
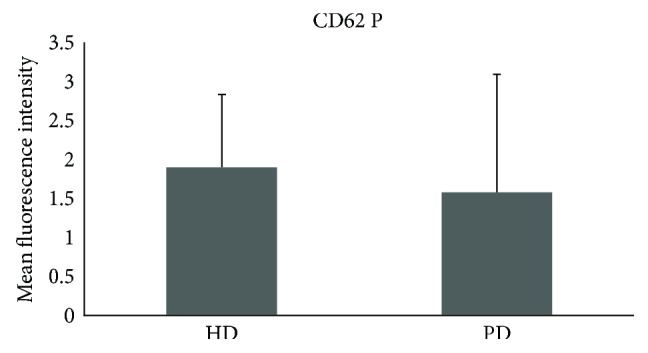
Significant difference between HD and PD on surface expression of CD62 P on platelets (*p* = 0.017). HD = haemodialysis; PD = peritoneal dialysis.

**Figure 2:**
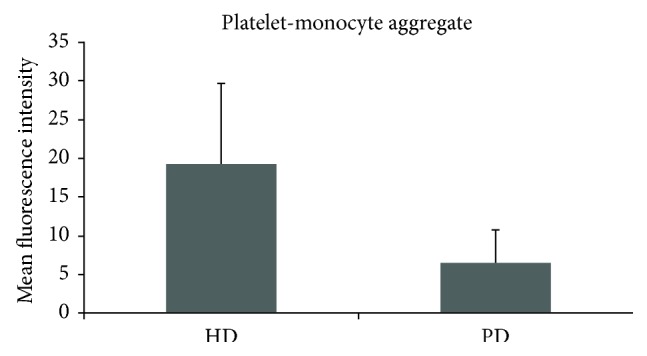
Significant difference between HD and PD on platelet-monocyte aggregates (*p* < 0.0001). HD = haemodialysis; PD = peritoneal dialysis.

**Figure 3:**
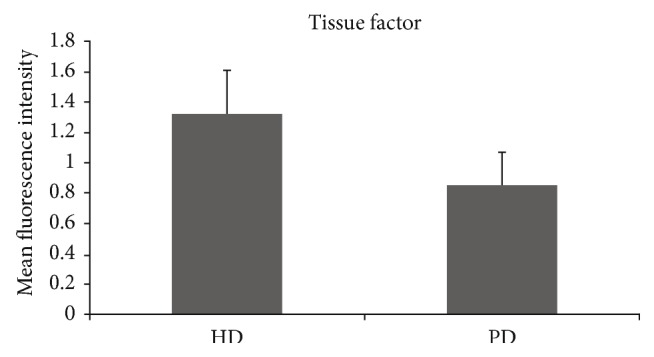
Significant difference between HD and PD on surface expression of tissue factor on monocytes (*p* < 0.0001). HD = haemodialysis; PD = peritoneal dialysis.

HD patients had significantly higher CD62P expression on platelets compared to PD patients (1.9 ± 0.9 versus 1.6 ± 1.5; *p* = 0.017) (Figure 1). We also found a significantly higher amount of platelet-monocyte aggregates (19.3 ± 10.4 versus 6.6 ± 4.2; *p* < 0.0001) (Figure 2) and tissue factor binding on monocytes (1.3 ± 0.3 versus 0.9 ± 0.2; *p* < 0.0001) in HD patients compared to that in PD patients (Figure 3). There was no significant difference between both patient groups regarding platelet surface expression of CD40L.

There was no significant correlation between Kt/V and any of the determined parameters in HD patients (data not shown). In PD patients, we found a significant correlation between Kt/V and CD40L (*r* = 0.867; 0.001) and between Kt/V and CD62P (*r* = 0.686; *p* = 0.028), while there was no significant correlation between Kt/V and tissue factor binding on monocytes and platelet-monocyte aggregates, respectively (data not shown).

### 3.2. Correlation of Weekly Dialysis Duration with Expression of Tissue Factor

**Figure 4:**
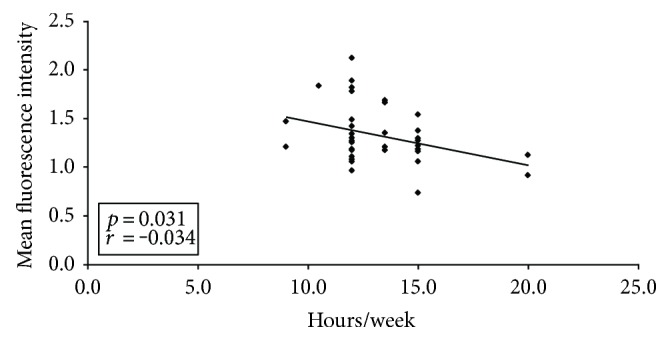
The expression of tissue factor on monocytes was significantly decreased in comparison to increasing time of haemodialysis (*p* = 0.031). ♦ indicates tissue factor.

The expression of tissue factor on monocytes was significantly decreased in comparison to increasing duration of haemodialysis sessions (*p* = 0.031) (Figure 4).

### 3.3. Effect between Haemodialysis and Peritoneal Dialysis on Cytokine Production

**Figure 5:**
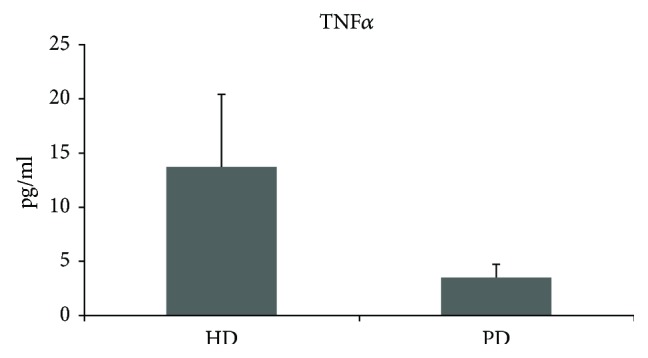
Significant difference between HD and PD on cytokine production of TNF*α* (*p* = 0.019). HD = haemodialysis; PD = peritoneal dialysis.

HD patients had significantly higher cytokine production of TNF*α* compared to PD patients (9.25 ± 11.13 versus 3.56 ± 1.24; *p* = 0.019) (Figure 5). There were no significant differences between both patient groups regarding cytokine production of IL-6, MCP-1, and sCD40L.

## 4. Discussion

Patients with end-stage renal disease (ESRD) undergo renal replacement therapy to avoid fatal complications of uremic toxins, elevated potassium level, and over hydration. Unfortunately, ESRD is conjoined with increased atherosclerotic and thromboembolic morbidity and mortality. In Germany, renal replacement therapy involves mostly haemodialysis and in a smaller proportion peritoneal dialysis treatments.

Therefore, the aim of this study was to investigate a possible difference between HD and PD with respect to the expression of platelet and monocyte activity markers and the tissue factor system. Numerous authors have previously addressed the question of platelet and coagulation activation in haemodialysis patients [16–18]. One important finding is that the presence of cardiovascular disease is closely linked to the tissue factor system [19]. Yorioka et al. described higher levels of tissue factor on monocytes in haemodialysis patients in comparison to a healthy control group [14]. Pawlak et al. demonstrated a relationship between increased oxidative stress and elevated tissue factor in peritoneal dialysis patients [20]. Al-Saady et al. showed a hypercoagulable state in patients with chronic renal failure as possible explanation for the increased occurrence of cardiovascular and cerebrovascular events [21]. Hypercoagulation being higher in patients on peritoneal dialysis than haemodialysis patients has been reported by Malyszko et al. [18]. The present study showed a significantly elevated expression of tissue factor on monocytes in haemodialysis patients in comparison to peritoneal dialysis patients. Referring to this, dialysis duration seems to be alleviating since haemodialysis duration was positively correlated with decreased tissue factor expression on monocytes in our study. This may explain that frequent haemodialysis was associated with favourable changes in reduction of left ventricular mass [22].

Actually, platelet and monocyte activity markers of atherosclerosis seem to be higher in haemodialysis patients than in peritoneal dialysis patients. In this study, the expression of CD62P on platelets in haemodialysis patients was significantly higher in comparison to that in peritoneal dialysis patients. Scialla et al. evaluated the positive association between soluble P-selectin and atherosclerotic cardiovascular disease in dialysis patients [23]. Especially, male patients showed significantly higher levels of soluble P-selectin and increased cardiovascular disease. Furthermore, this study underlines previous results of Ashman et al. [24] with regard to an increased activation of platelets in dialysis patients, demonstrating more platelet-monocyte aggregates in haemodialysis patients in comparison to those in peritoneal dialysis patients.

Another attempt to reduce cardiovascular disease is to target classical cardiovascular risk factors. In this line, surprisingly, the 4D and AURORA study showed no beneficial effect for lipid-lowering strategies in diabetic and nondiabetic haemodialysis patients [25, 26]. In our study, a higher proportion of PD patients received statins. This could be a limitation of our study results, since potential anti-inflammatory properties of statins could have attenuated platelet and monocyte activity markers. In contrast to the negative results in clinical trials in haemodialysis patients, simvastatin reduced increased soluble CD40L levels in peritoneal dialysis patients [27]. Expression of CD40L on platelets in this study however did not differ significantly between haemodialysis and peritoneal dialysis patients. Schwabe et al. demonstrated elevated levels of sCD40L in patients with chronic renal disease in comparison to healthy controls [28]. This and other studies raise the question whether dialysis modality may influence cardiovascular risk and mortality [29–31]. In the DOC study, after correction for possible confounders, no statistically significant difference was found in mortality between HD and PD patients [32]. Similarly, no difference was found for the de novo development of coronary heart disease [33]. But there exist potential differences between HD and PD in influencing cardiovascular risk factors, which are altered by uremic toxins [34], fluid overload, and inflammation [35]. Indeed, prior publications are heterogeneous in valuating possible advantages and disadvantages of the two dialysis modalities HD and PD [29–31].

Several toxins such as urea contribute to impaired platelet function and are cleared by dialysis. Serum levels of these dialysable substances still do not correlate with bleeding time or platelet adhesion [36]. The net effect, however, is a reduction of platelet reactivity and an enhanced formation of platelet-dependent fibrin clots contributing to the improved platelet function after dialysis.

We provide evidence that cytokine plasma levels of TNF alpha are significantly higher in haemodialyis patients compared to those in peritoneal dialysis patients, but no significant differences in the other cytokines assessed were detectable.

In peritoneal dialysis, platelet hyperactivity may occur due to common hypoalbuminemia and dyslipidaemia [37]. On the other hand, the higher GPIIb/IIIa expression, a lack of systemic heparinization, an improved clearance of “middle molecules,” and the absence of the artificial surfaces of the dialyser and blood tubing account for a lower risk of haemorrhage [38, 39]. It is known that a disturbed platelet function is an important cause for thromboembolic and haemorrhagic complications in ESRD patients.

Dialysis therapy is the therapeutic standard in patients with ESRD but cardiovascular morbidity and mortality remain unacceptably high in such patients. In the present study, platelet and monocyte activity markers of atherosclerosis seem to be higher in HD patients in comparison to PD patients, possibly suggesting a higher risk of atherosclerotic morbidity and mortality in this major subgroup of dialysis patients.

## Figures and Tables

**Table 1:** Study population.

	HD patients *n* = 41	HD patients (%)	PD patients *n* = 10	PD patients (%)	*p* value
Age (years)	61.83 ± 15.42		53.2 ± 11.25		0.126
Male	25	60.98	6	60	1
Hyperlipidemia	6	14.63	6	60	**0.007**
Coronary artery disease	12	29.27	2	20	0.707
Peripheral arterial disease	8	19.51	2	20	1
Diabetes mellitus	15	36.59	1	10	0.142
Hypertension	35	85.37	10	100	0.331

**Table 2:** Medication in use at baseline.

Medication	HD patients *n* = 41	HD patients (%)	PD patients *n* = 10	PD patients (%)	*p* value
ASA	5	12.2	1	10	1
Oral anticoagulants	9	21.95	1	10	0.664
Beta blocker	22	53.66	5	50	1
Statins	4	9.76	4	40	**0.038**
ACE-inhibitor/AT(1) antagonist	20	48.78	6	60	0.727

ASA = acetylsalicylic acid; ACE inhibitor = angiotensin-converting-enzyme inhibitor; AT (1) antagonist = angiotensin (1) antagonist.
